# Isolated Thoracic Anterior Spinal Artery Aneurysm in a Patient With Autoimmune Disease: A Case Report

**DOI:** 10.7759/cureus.64943

**Published:** 2024-07-19

**Authors:** Arjang Ahmadpour, Rami Z. Morsi, Mohammed Maan Al-Salihi, Tareq Kass-Hout

**Affiliations:** 1 Department of Neurosurgery, University of Chicago, Chicago, USA; 2 Department of Neurology, University of Chicago, Chicago, USA; 3 Department of Neurological Surgery, School of Medicine and Public Health, University of Wisconsin, Madison, USA

**Keywords:** thoracic spine, systemic mycosis, subarachnoid hemorrhage, autoimmune, aneurysm

## Abstract

This case report describes a rare presentation of a mycotic anterior spinal artery aneurysm of the thoracic spine presenting as a subarachnoid hemorrhage. Isolated anterior spinal artery aneurysms are exceedingly rare. While this condition can occur in the setting of an underlying infection that may lead to shock, other signs and symptoms of the infection itself typically manifest before the development of the aneurysm and subsequent hemorrhage. We present a case of a 30-year-old male who presented with acute-onset bilateral lower extremity motor paraplegia and was found to have diffuse subarachnoid hemorrhage related to an isolated thoracic anterior spinal artery aneurysm, which was believed to be mycotic in origin. Spinal angiogram revealed evidence of an aneurysm originating from the anterior spinal artery at the T11-T12 level, contributing to diffuse subarachnoid hemorrhage of the spinal cord. The patient was followed closely and exhibited progressive improvement in motor function. Magnetic resonance imaging performed two weeks later revealed decreased intrathecal hemorrhage, mild spinal cord edema, and a reduction in the discrete visualization of the anterior spinal artery aneurysm. We present a unique case of an isolated anterior spinal artery aneurysm in the thoracic spine presenting with subarachnoid hemorrhage. This case is distinctive in that the clinical presentation and radiographic findings strongly suggest a mycotic etiology for the aneurysm, despite the absence of definitive histopathologic confirmation. To our knowledge, this is the first reported case of an isolated thoracic ASA aneurysm suspected to be mycotic in origin.

## Introduction

We report a case of a ruptured isolated anterior spinal artery (ASA) aneurysm of the thoracic spine in a patient with autoimmune disease and systemic polymicrobial infection. Isolated ASA aneurysms are exceedingly rare vascular lesions and can be associated with arteriovenous malformations and arteriovenous fistulas, but they can also occur in the setting of hemodynamic stress [1-3]. There have been few reports of thoracic ASA aneurysms presenting with SAH [4-6]. This is the first report of an isolated ASA aneurysm of the thoracic spine presenting with SAH, strongly believed to be of mycotic origin.

## Case presentation

A 30-year-old male with a past medical history of COVID-19 infection, cytomegalovirus-associated sepsis, and atypical hemolytic uremic syndrome (aHUS), as well as concern for mixed connective tissue disease with positive anti-U1 ribonucleoprotein antibodies, presented to the hospital with fatigue and shortness of breath. On hospital day 1, the patient was evaluated for acute-onset heart failure with uncontrolled high blood pressure, requiring continuous clevidipine infusion. On hospital day 9, the patient had acute-onset back pain and bilateral lower extremity weakness with preserved sensation. On examination, he was found to have bilateral lower extremity motor paraplegia, no rectal tone, and no sensory deficits. He reported no neck stiffness or pain. On day 11, he required urgent hemodialysis due to acute renal failure in the setting of hypertensive emergency.

**Figure 1:**
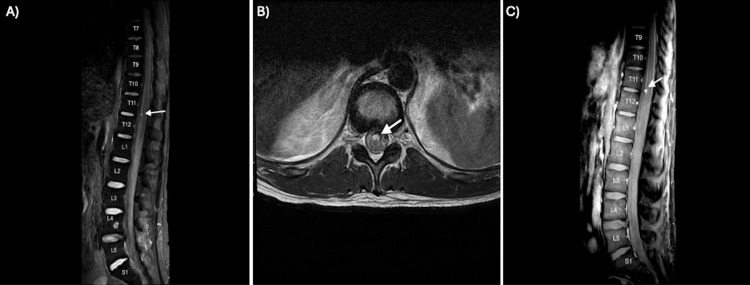
MRI of the thoracolumbar spine with STIR sequence (A), axial T2-weighted sequence (B), and post-contrast T1-weighted sequence (C) showing diffuse abnormal signal through subarachnoid space with 6mm enhancing lesion noted anteriorly in the thecal sac at the T11-T12 level (noted as an arrow). MRI, magnetic resonance imaging; STIR, short T1-inversion recovery

The differential diagnosis at the first onset of symptoms included spinal cord infarct (given pure motor paraplegia in the lower extremities), spinal lesion, or an autoimmune process (such as a demyelinating disease or transverse myelitis). Computed tomography (CT) of the head showed no acute intracranial abnormalities. Magnetic resonance imaging (MRI) of the complete spine showed diffuse abnormal signal throughout the subarachnoid space of the spine, most prominent in the lower thoracic spine (Figure 1A). A T2-hyperintense and enhancing lesion within the thecal sac at T11-T12 was also seen (Figures 1B, 1C, respectively).

**Figure 2:**
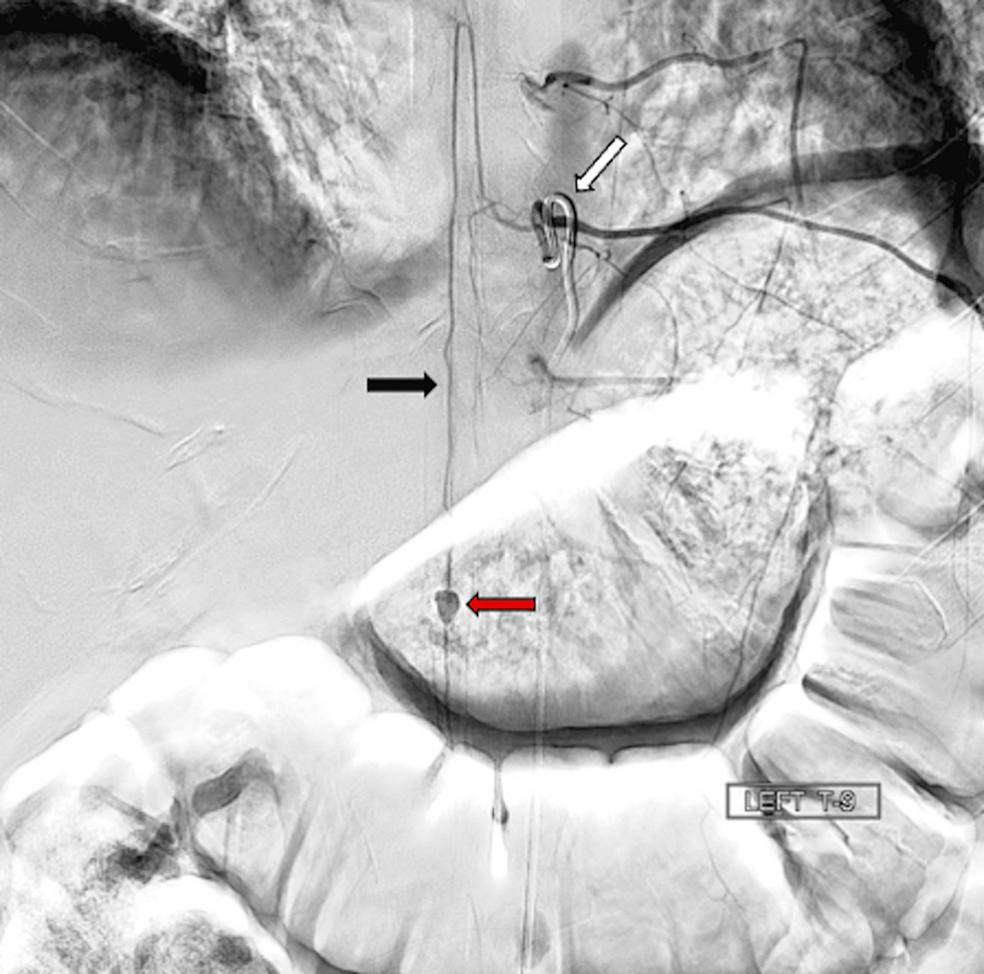
Diagnostic spinal angiogram showing the artery of Adamkiewicz visualized from the left T9 level (white arrow), and anterior spinal artery flowing down to the conus medullaris level (black arrow) with evidence of an aneurysm originating from the anterior spinal artery itself measuring 7 mm by 5 mm (red arrow).

Spinal angiogram revealed a 7 x 5 mm ASA aneurysm at the T11-T12 level (Figure 2). The patient was closely observed without any intervention, as therapeutic occlusion of the aneurysm could not be undertaken without risking spinal cord infarction at that time, with plans for repeat surveillance imaging.

**Figure 3:**
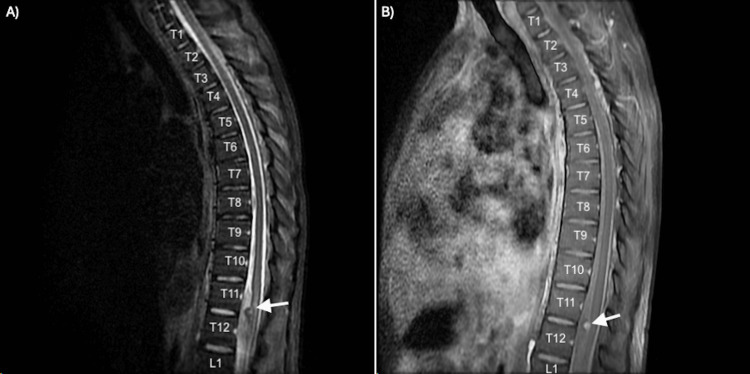
MRI of the thoracic spine with STIR sequence (A) displaying edema at the conus medullaris level adjacent to the site of the aneurysm and post-contrast T1-weighted sequence (B) showing enhancement of the aneurysm (aneurysm noted with an arrow). MRI, magnetic resonance imaging; STIR, short T1-inversion recovery

The patient exhibited progressive improvement in motor function. Repeat MRI of the thoracolumbar spine showed edema at the conus medullaris level and stable aneurysm (Figure 3).

**Figure 4:**
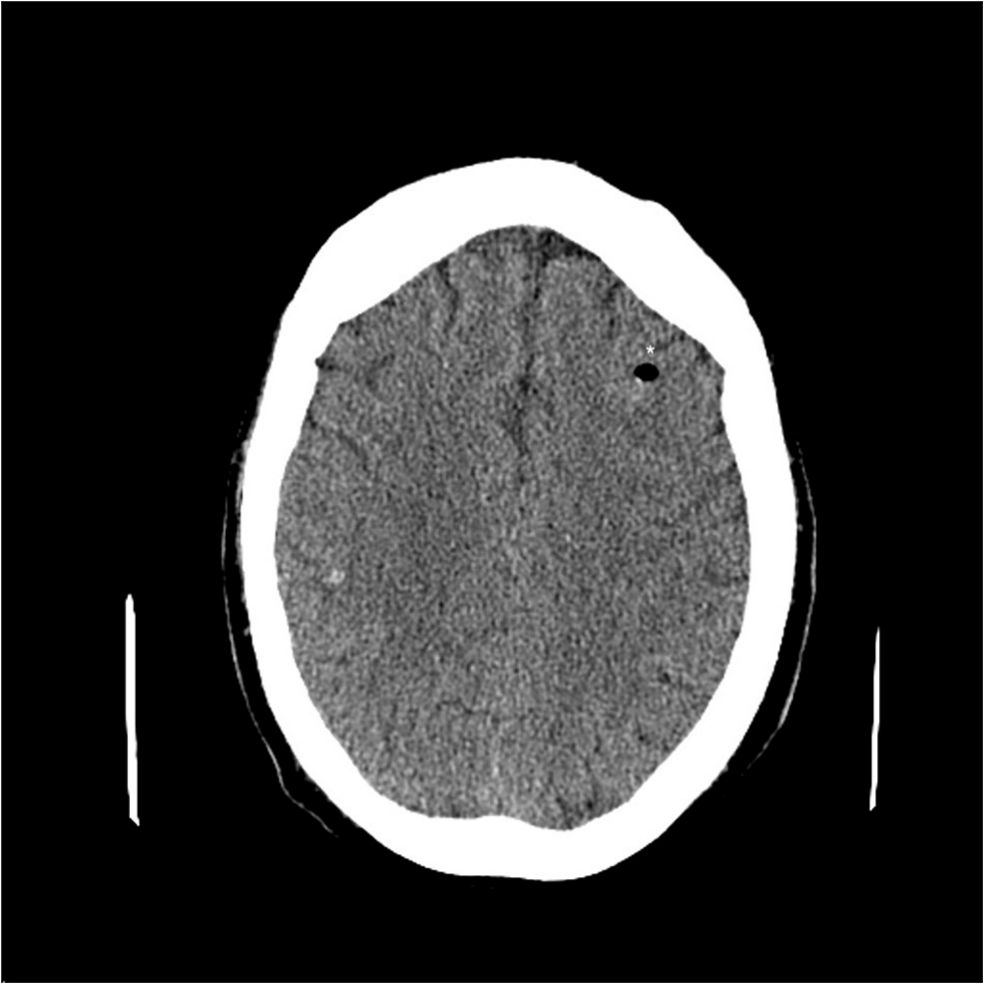
Computed tomography scan of the head showing hyperdense foci near gray-white junction in the left frontal and right parietal lobes with left frontal focus of pneumocephalus (noted as an asterisk “*”), highly suspicious for septic emboli.

Two months following hospital discharge, the patient developed recurrent chest pain and shortness of breath. He received emergent right heart catheterization, which showed acute heart failure. An intra-aortic balloon pump was placed, and extracorporeal membrane oxygenation (ECMO) was initiated. The patient also became pancytopenic with a white blood cell count of 3.4 x 10^3^/μL, platelet count of 54 x 10^3^/μL, and a hemoglobin level of 8.8 g/dL. Differential diagnosis at this stage included septic shock, hemolytic anemia, antiphospholipid syndrome, and hemophagocytic lymphohistiocytosis (HLH). The patient was considered for disseminated intravascular coagulation, but fibrinogen level was normal and there were no schistocytes on peripheral blood smear. He was noted to have positive anticardiolipin IgG autoantibody, but repeat testing did not reveal confirmation of any autoantibodies associated with antiphospholipid syndrome. The patient met only four of the following criteria for HLH: elevated ferritin level (4,498 ng/mL), pancytopenia, hypertriglyceridemia (504 mg/dL), and fever. The patient was started on continuous therapeutic-intensity heparin infusion and intravenous hydrocortisone while on ECMO. Medical workup revealed bacteremia, with blood cultures that grew *Streptococcus mitis*, *Granulicatella adiacens*, and methicillin-susceptible *Staphylococcus aureus*. CT of the chest showed interval patchy multifocal cavitary airspace disease, suggestive of necrotizing pneumonia. On hospital day 2, the patient demonstrated a decline in neurological examination and stopped following commands. CT of the head at that time showed no abnormalities. On hospital day 5, the patient developed fixed and dilated pupils, with no movement of his extremities. Repeat CT of the head showed hyperdense foci near the gray-white junction within the left frontal and right parietal lobes, with a focus on pneumocephalus on the left, concerning for septic emboli with possible abscess formation (Figure 4).

CT angiogram of the head showed diffuse cerebral edema with reduced intracranial circulation. Given these findings, the family withdrew care. The patient died soon thereafter. Autopsy was performed, and the cause of death was attributed to complications from severe sepsis secondary to necrotizing pneumonia. This was attributed to his underlying autoimmune disease and thrombotic microangiopathy. Microscopic examination of the heart revealed patchy interstitial fibrosis, suggestive of a healed myocarditis. The lungs showed multiple abscess cavities, numerous thrombi, endothelial proliferation, and intimal fibrosis of the pulmonary vasculature. The brain showed diffuse edema, multiple white matter hemorrhages, and hypoxic-ischemic injury. Diffuse cerebral edema was attributed to a high level of inflammatory cytokines from sepsis. These findings were supportive of an infectious etiology as the source of the patient’s thoracic ASA aneurysm, although no final pathology or culturing of the vessel was performed at the time of autopsy. Informed consent was obtained from the family to publish these findings. Infections, such as COVID-19, can manifest more severely in patients with aHUS by causing increased complement activation and endothelial injury, leading to end-organ damage [4]. Genetic variations in complement regulation have shown that susceptibility to serious complications can persist long after the initial infection has resolved [7].

## Discussion

ASA aneurysms are typically heterogenous in anatomic location, morphology, and pathophysiology, and can be related to conditions contributing to hemodynamic stress through the arteries [2]. Isolated spinal artery aneurysms are not associated with hemodynamic stress and instead are believed to be related to conditions contributing to inflammation of the arterial wall, which can lead to dissection of the vessel [8]. A recent review of 107 cases of isolated aneurysms of the spinal circulation showed that ASA aneurysms are most commonly seen in patients with aortic coarctation [9]. Other proposed mechanisms of spinal artery aneurysm include mechanical injury of the artery itself, Valsalva maneuvers, connective tissue disorders, autoimmune diseases, Moya Moya disease, or a congenital origin [9]. No previous reviews have demonstrated an infectious etiology causing ASA. Most patients with isolated spinal artery aneurysms are female, with half of the cases localized to the thoracic spine. The most involved artery was the ASA, followed by the posterior spinal artery, the radiculopial arteries, the radiculomedullary arteries, and radicular branches. Most isolated spinal artery aneurysms are fusiform as they tend to occur along the course of the spinal arteries without involving the branching points [9,10].

The most common clinical presentation of ruptured spinal artery aneurysms is back pain. Subarachnoid hemorrhage is seen with imaging in approximately 80% of cases. Ruptured spinal artery aneurysms can also present with intracranial symptoms such as headache, emesis, and photophobia [10,11]. The aneurysm formation in our patient might have been related to the inflammatory state caused by aHUS via complement overactivation and acquired autoantibody formation. Berlis et al. describe the case of a male patient who was noted to have paraplegia and was found to have a contrast-enhancing lesion found to be an aneurysm of the artery of Adamkiewicz, thought to be related to a systemic Candida infection [12]. Our patient had systemic polymicrobial infection detected after diagnosis of the ASA aneurysm. The rupture of the aneurysm was likely a result of the fragility of the arterial wall.

The pathophysiology contributing to isolated ASA formation remains unclear. This is due to both a dearth of reported cases and a lack of animal models that adequately simulate human disease. Therefore, proposed mechanisms in the literature are mostly attributed to conjecture from expert opinions [10]. Spinal aneurysm histopathology is poorly reported in the literature. Microscopically, spinal aneurysms show outpouching of a segment of the vessel. These areas have shown thrombus formation composed of scattered leukocytes, lytic erythrocytes, and fibrin surrounded by loose bundles of collagen fibers without muscular tunica media [10]. Histologic signs of inflammation and myxoid degeneration might also be seen [8,13]. Other reports show that spinal artery aneurysms may be congenital in origin [14-16]. Histopathological examination of congenital aneurysms has shown vessel wall weakening secondary to an absence of elastic lamina, resulting in saccular aneurysmal dilation [10,14-16].

The overall clinical and radiographic characteristics for reported cases of thoracic ASA aneurysms from the literature are summarized in Table 1.

**Table 1:** Reported cases of anterior spinal artery aneurysms at the thoracic level

Study ID	Patient No.	Age/ Sex	Location (level)	Clinical presentation	Evidence of rupture	Treatment	Clinical Outcomes	Radiographic Outcomes
Ahmadpour et al. (present case)	1	30/M	T11-T12	Acute-onset back pain, bilateral lower extremity weakness	Yes	Conservative	Death (due to septic shock)	Diffuse cerebral edema with reduced intracranial circulation
Cobb et al., 2020 [17]	2	36/M	T11	Left-sided abdominal pain, nausea, and vomiting	Yes	Endovascular approach (Onyx embolization)	No signiﬁcant clinical improvement	Complete occlusion/resolution
Dabus et al., 2017 [18]	3	63/F	Lower thoracic	Low back pain	Yes	Conservative	Complete recovery	Complete occlusion/resolution
El Mahdi et al., 1989 [19]	4	17/F	T12	Back pain, bladder dysfunction	No	Surgery (clipping of the feeding artery)	Complete recovery	Complete occlusion/resolution
Garcia et al., 1979 [16]	5	34/F	T6	Chest pain, meningismus, weakness	Yes	Conservative	Death	Not reported
Gonzalez et al., 2005 [20]	6	73/M	T6-T7	Back pain, headache	Yes	Surgery (resection)	Partial recovery	Surgical occlusion
Gonzalez et al., 2005 [20]	7	54/M	T12	Back pain, radicular pain	Yes	Surgery (resection)	Complete recovery	Surgical occlusion
Gutierrez Romero et al., 2014 [5]	8	37/F	T3	Thoracic pain, cervical pain, headache, meningismus	Yes	Conservative	Complete recovery	Spontaneous resolution
Kito et al., 1983 [21]	9	37/F	T10	Meningismus, back pain, bladder dysfunction	Yes	Conservative	Partial recovery	Not reported
Lavoie et al., 2007 [6]	10	12/M	T8	Headache, vomiting	Yes	Endovascular approach (coil embolization)	Complete recovery	Complete occlusion/resolution
Leech et al., 1976 [14]	11	25/F	T7	Back pain, weakness, bladder and bowel dysfunction	No	Surgery (resection)	Partial recovery	Complete occlusion/resolution
Ling and Bao, 1994 [8]	12	14/M	Upper thoracic	Headache, loss of consciousness, paraplegia, bowel and bladder incontinence, sensory level	Yes	Surgery (aortic bypass with artificial vessel graft)	Partial recovery	Not reported
Longatti et al., 2008 [22]	13	54/F	T9–12	Abdominal pain, vomiting, meningismus, leg weakness	Yes	Conservative	Complete recovery	Spontaneous resolution
McGuire et al. 2023 [23]	14	23/M	T7	Neck pain, paraplegia, sensory level	Yes	Endovascular approach (glue embolization) and surgery (laminectomy for hematoma evacuation)	Partial recovery (ambulatory)	Decrease in mass effect
McGuire et al. 2023 [23]	15	54/M	T12	Neck pain, back pain, paraplegia	Yes	Surgery (laminectomy for surgical trapping with hematoma evacuation)	Partial recovery (ambulatory)	Surgical occlusion/resolution
McGuire et al. 2023 [22]	16	60/F	T3, T6, T10	Lower extremity weakness	Yes	Conservative	Complete recovery (ambulatory)	Spontaneous regression of three aneurysms with resolution of the fusiform dilatations of the radiculomedullary arteries
McGuire et al. 2023 [23]	17	64/M	T8, T10	Back pain, urinary retention	Yes	Conservative	Complete recovery (ambulatory)	Resolution of both aneurysms
Nguyen et al., 2021 [24]	18	45/M	T9	Abdominal pain, followed by severe headache, vomiting, and generalized seizure	Yes	Surgery (laminoplasty and microsurgical resection)	Partial recovery	Surgical occlusion/resolution
Rengachary et al., 1993 [25]	19	50/F	T12	Back pain	Yes	Surgery (resection)	Partial recovery	Not reported
Seerangan et al., 2012 [26]	20	47/M	T7-T10	Lower extremity weakness, bowel and bladder dysfunction	Yes	Surgery (resection)	Partial recovery (rehabilitation)	Not reported
Smith et al., 1986 [27]	21	29/M	T12	Back pain, meningismus	Yes	Surgery (clipping with clot evacuation)	Partial recovery	Not reported
Smith et al., 2019 [28]	22	52/F	T11	Back pain, ascending lower extremity weakness	Yes	Surgery (laminectomy and surgical exploration)	Partial recovery	Suspicious small aneurysms leading to the diagnosis of polyarteritis nodosa
Sung et al., 2015 [29]	23	74/M	T1-T2	Chest pain radiating to the neck and back, headache, confusion	Yes	Surgery (laminectomy and surgical exploration)	Partial recovery	Surgical occlusion/resolution
Vishteh et al., 1997 [30]	24	30/M	T11	Headache, back pain, bilateral lower extremity paresthesias	Yes	Surgery (wrapping with muslin gauze and clip reinforcement)	Complete recovery	Not reported

There were 24 cases (including the present case) mainly related to thoracic ASA aneurysms, 14 men and 10 women, with age ranging from 12 years to 74 years (mean [± SD]: 42 ± 18 years). These aneurysms occurred predominantly in the lower thoracic levels (T10-T12). Aneurysms were treated with surgery, including endovascular techniques, in 16 cases. Complete recovery was achieved in nine cases, partial recovery in 12 cases, no improvement in one case, and mortality occurred in two cases. Notably, our case resulted in death, which was believed to be due to sepsis with a high likelihood of mycotic aneurysm. While our case of believed mycotic aneurysm did not rebleed, mycotic aneurysms originating from the spine may be at increased risk of rebleeding, such as that seen in intracranial mycotic aneurysms [31]. Only one other case of a mycotic radiculomedullary artery aneurysm at the C5 level was found, and this patient required surgical intervention due to neurological status worsening as a result of rebleeding [32]. Mycotic aneurysms typically result from an inflammatory response triggered by a bacterial infection. The bacterial infection draws neutrophils into the arterial wall, by way of cytokines, and matrix metalloproteinases become activated and contribute to infiltration and vessel wall dilatation and eventually aneurysmal rupture [33].

## Conclusions

Mycotic aneurysms, especially in the spine, are uncommon and should be suspected on a case-by-case basis in patients with active infections or autoimmune pathologies. Isolated ASA aneurysms are rare, can present with spinal SAH, and can be associated with systemic mycosis. It is important to include a comprehensive medical evaluation when spinal artery aneurysms are discovered, as these aneurysm types might be attributed to a systemic inflammatory process, as well as the more traditional etiologies that include various physiological factors, for early diagnosis and treatment. Clinical implications of this case warrant a higher index of suspicion for mycotic aneurysms especially in immunocompromised patients, a multidisciplinary approach involving neurosurgeons, radiologists, and infectious disease specialists, longer-term follow-up imaging, and treatment typically involving a combination of antimicrobial therapy, surgical or endovascular intervention, and supportive care.
